# Epibrassinolide improves the growth performance of *Sedum lineare* upon Zn stress through boosting antioxidative capacities

**DOI:** 10.1371/journal.pone.0257172

**Published:** 2021-09-07

**Authors:** Yiyang Zhang, Hui Liao

**Affiliations:** Shanghai High School International Division (SHSID), Shanghai, China; Government College University Faisalabad, PAKISTAN

## Abstract

As an essential element, zinc (Zn) can improve or inhibit the growth of plants depending on its concentrations. In this study, the effects of 24-Epibrassinolide (EBR), one well-known steroid phytohormone regulating plant growth and alleviating abiotic stress damage, on morphological parameters and antioxidant capacities of *Sedum lineare* were investigated under different Zn doses. Compared to plants only exposed to Zn, simultaneously foliar application of 0.75 μM EBR significantly improved multiple morphological characteristics and such growth-improving effects were more significant at high Zn concentrations. At a detrimental 800 μM Zn, EBR benefitted plant growth most prominently, as shown by that the stem length, fresh weight and internode length were increased by 111%, 85% and 157%, respectively; than Zn solely treated plants. EBR spray also enhanced both the activities of antioxidant enzymes such as peroxidase (POD), ascorbate peroxidase (APX), glutathione reductase (GR), and the contents of antioxidative agents including ascorbic acid (AsA) and glutathione (GSH), which in turn decreased the accumulation of reactive oxygen species (ROS) and alleviated the lipid peroxidation in plants. Thus, by demonstrating that EBR could help *S*. *lineare* resist high-zinc stress through strengthening the antioxidant system, this work provided a new idea for expanding the planting range of *Crassulaceae* plants in heavy metal contaminated soil for phytoremediation purpose in the future.

## Introduction

As one of the well-known essential micronutrients and a cofactor for various enzymes involved in metabolic activities, Zinc is directly involved in the growth and development of plants [1, 2]. Under normal conditions, soil contains a low level of zinc; however, industrial processes such as mine tailings, smelting, waste disposal or leakage and the use of industrial wastewater for agricultural irrigation have greatly increased the presence of zinc in agricultural soil, and excessive zinc could lead to stem growth retardation, root development inhibition and morphological/anatomical leaf changes [3–5]. On the cellular level, excessive zinc will induce the generation of reactive oxygen species (ROS), cause oxidative damage to nucleic acids, pigments, proteins and membrane lipids, change antioxidant enzyme activity and affect the level of antioxidants in plant cells, thereby disturb the cell’s redox homeostasis [6].

As a class of steroids that play key roles in plant growth and development, brassinosteroids (BRs) are actively involved in a variety of physiological processes [7, 8]. BRs have been proven to enhance the activity of antioxidative enzymes, increase the content of non-enzymatic antioxidants and reduce the accumulation of ROS and MDA in order to defend plants against various abiotic stresses [9, 10]. BRs have also been demonstrated to significantly improve the tolerance of mustard and chickpea under cadmium, aluminum, and nickel stresses [10, 11]. 24-Epibrassinolide (EBR) is one of the most biologically active forms of naturally occurring BRs [12–14] and its exogenous treatment has been shown to improve plant metabolism and increase the fresh biomass [15–17].

*S*. *lineare* is a perennial herb of *Crassulaceae*, it has succulent stems and is mainly used for roof greening in garden applications due to its easy cultivation and management. Plus, *S*. *lineare* is commonly regarded as a pioneer plant showing excellent resistances to water stress, drought stress and salt stress [18], which makes it an intriguing system to explore the complex defense mechanisms of plants [19]. In this paper, we studied the morphological and physiological characteristics of *S*. *lineare* under different zinc stresses with or without exogenously applied EBR with an emphasis on investigating the involvement of ROS elimination mechanism in the mitigating effects of EBR on zinc toxicity.

## Materials and methods

### Plant materials, growth conditions and treatments

*S*. *lineare* seedlings were grown in pots with a diameter of 8 cm filled with sterilized-sand and vermiculite (3:1) for seven days in a growth chamber at 24°C with 65% relative humidity and 16 h/8 h light regime. After 7 days, plants of one group were watered with 1/2 Hoagland’s nutrient solution containing with different concentrations of zinc sulfate as 0, 20, 200 and 800 μM, respectively; and plants of the second group were treated in the same manner except for being simultaneously sprayed with 0.75 μM EBR every 8 hours. Each group was set up for at least 20 plants and the experiment was carried out using 3 independent biological replicates. The stem length, internode length and plant fresh mass were recorded at day 14.

### 3,3ʹ-diaminobenzidine (DAB) and nitroblue tetrazolium (NBT) staining as well as malondialdehyde (MDA) determination

The fresh leaves were immersed in DAB staining solution (4.67 mM DAB, 1% isopropanol and 0.1% Triton X-100) and NBT staining solution (0.5 mg/ml NBT and 10 mM NaN_3_, 10 mM potassium phosphate buffer, pH 7.8) for 12 h to detect the presence of H_2_O_2_ and O_2_^•−^, respectively [20]. The reactions were stopped by transferring the leaves into absolute ethanol heated in a boiling water bath for 10 minutes to remove chlorophyll. Finally, the samples were put on a paper towel impregnated with 60% glycerin for photo observation. For MDA estimation, 0.5 mL plant supernatant was mixed with 2 mL of 20% (v/v) trichloroacetic acid (TCA) containing 0.5% (w/v) thiobarbituric acid (TBA) and then reacted in a boiling water bath for 30 min before the absorbance of the supernatant was then recorded at A_532_, A_600_ and A_440_, respectively [21].

### Extraction and activity measurements of antioxidant enzymes

For activity measurements of antioxidant enzymes, 0.5 g fresh tissues (leaves or roots) were homogenized rapidly in liquid nitrogen and suspended in 3 mL 50 mM phosphate buffer (pH 7.0) containing 0.1% Triton X-100 and 1% polyvinylpyrrolidone (w/v) [22]. After centrifugation at 12,000 g and 4°C for 15 min, the supernatant fraction was used for antioxidant enzyme activity assays.

As previously described, ascorbate peroxidase (APX, EC 1.11.1.11) activity was measured by monitoring the absorbance change at 290 nm (ε = 2.8 mM^-1^ cm^-1^) [23], peroxidase (POD, EC 1.11.1.7) activity was assayed by measuring the absorbance change due to oxidation of guaiacol at 470 nm (ε = 26.6 mM^−1^ cm^−1^) [24] and the activity of catalase (CAT, EC 1.11.1.6) was assayed by measuring the consumption of H_2_O_2_ at A_240_ (ɛ = 0.0394 mM^-1^ cm^-1^) [25]. The total superoxide dismutase (SOD, EC 1.15.1.1) activity was determined by the ability of SOD to inhibit the photochemical reduction of NBT and was calculated spectrophotometrically at A_560_ (ɛ = 100 mM^-1^ cm^-1^) [26]. Glutathione reductase (GR, EC 1.6.4.2) activity was measured by monitoring the glutathione-dependent oxidation of NADPH at 340 nm [27]. Protein was quantitated using BSA as a standard [28].

### Determination of antioxidant agent contents

To determine the contents of antioxidant agents, 0.5 g of fresh samples were ground in 6% trichloroacetic acid (TCA) and centrifuged at 3,000 g for 20 min. The extract reaction solution was then mixed with 0.5 mL Folin-phenol and determined at 760 nm for ascorbic acid (AsA) [29]. For Glutathione (GSH) measurement, 0.5 g plant tissue was ground to fine powder, homogenized in 1 mL of 3% TCA, centrifuged at 12,000 g, then measured for the absorbance at 412 nm [30].

### Statistical analysis

All the above data were analyzed by ANOVA with SPSS 20 (IBM Corporation, North Castle Drive, Armonk, NY, USA) statistical software and at least 3 biological replicates were set for each treatment. The data given is the mean ± standard deviation (SD) and Duncan test was used to make multiple comparisons of different treatments at the level of *P* < 0.05.

## Results and discussion

### Effects of zinc with or without EBR on shoot growth of *S*. *lineare*

**Fig 1:**
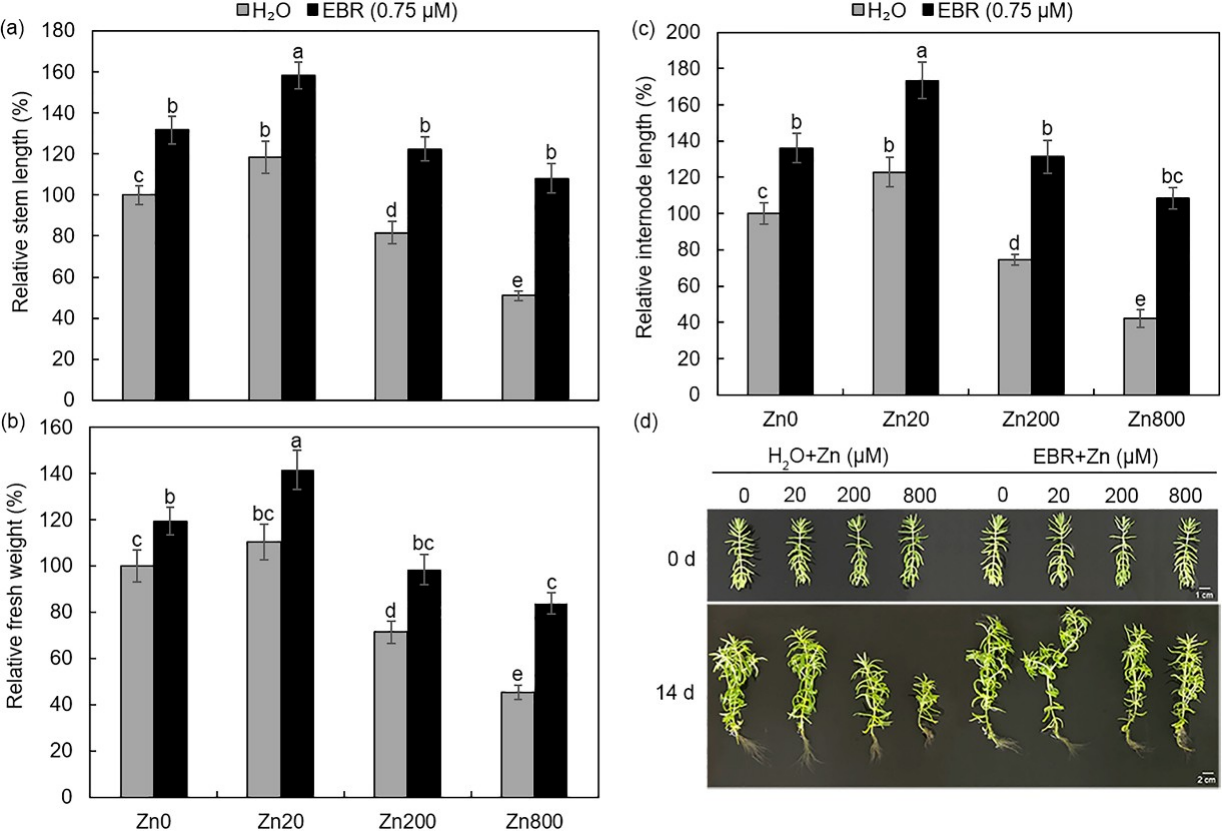
Effects of zinc and EBR treatments on growth indexes of *S*. *lineare* plants. **(a)** Relative stem length; **(b)** Relative fresh weight; **(c)** Relative internode length. **(d)** Photos of *S*. *lineare* treated with Zn and EBR for 14 d.

Through screening zinc concentrations in preliminary experiments, we confirmed the beneficial effect of 20 μM ZnSO_4_ (Zn20), and set 200 μM ZnSO_4_ (Zn200) as a toxicity threshold and 800 μM ZnSO_4_ (Zn800) as the dose causing serious adverse effects. Then, the dose-dependent responses of *S*. *lineare* to Zn at these three levels were evaluated with respect to stem length, internodal length and fresh weight. The average stem length of Zn20-treated plants were increased by 18%, while Zn200 and Zn800 decreased this index by 19% and 48%, respectively; as compared with that of untreated control plants (Fig 1A). Similar trends were also observed for other two growth indexes as shown in Fig 1B and 1C. Since zinc is an essential trace element and plays an important role in plant growth, the lower concentration of zinc promoted plant growth as expected [2, 31, 32], although that Zn25 has been reported to inhibit the growth of *Gossypium hirsutum* L. and Zn20 negatively impacted multiple physiological indexes of *Garlic* [33, 34], indicating that *S*. *lineare* is a fairly tolerant plant species against zinc stress.

We then screened EBR concentrations and identified 0.75 μM EBR as the beneficial dose with growth-promoting effects when exogenously applied to unstressed *S*. *lineare*. After 14 days of treatment, the stem length, fresh weight and internode length of EBR-sprayed plants were increased obviously (Fig 1), in agreement with the previous report that the application of exogenous BR could increase the shoot length, aerial part weight and the petiole extension of carrots [35]. Under Zn800, the beneficial effects of EBR on plant growth became even more prominent, as that the stem length, fresh weight and internode length were increased by 111%, 85% and 157%, respectively (Fig 1).

### Effects of zinc with or without EBR on root growth of *S*. *lineare*

**Fig 2:**
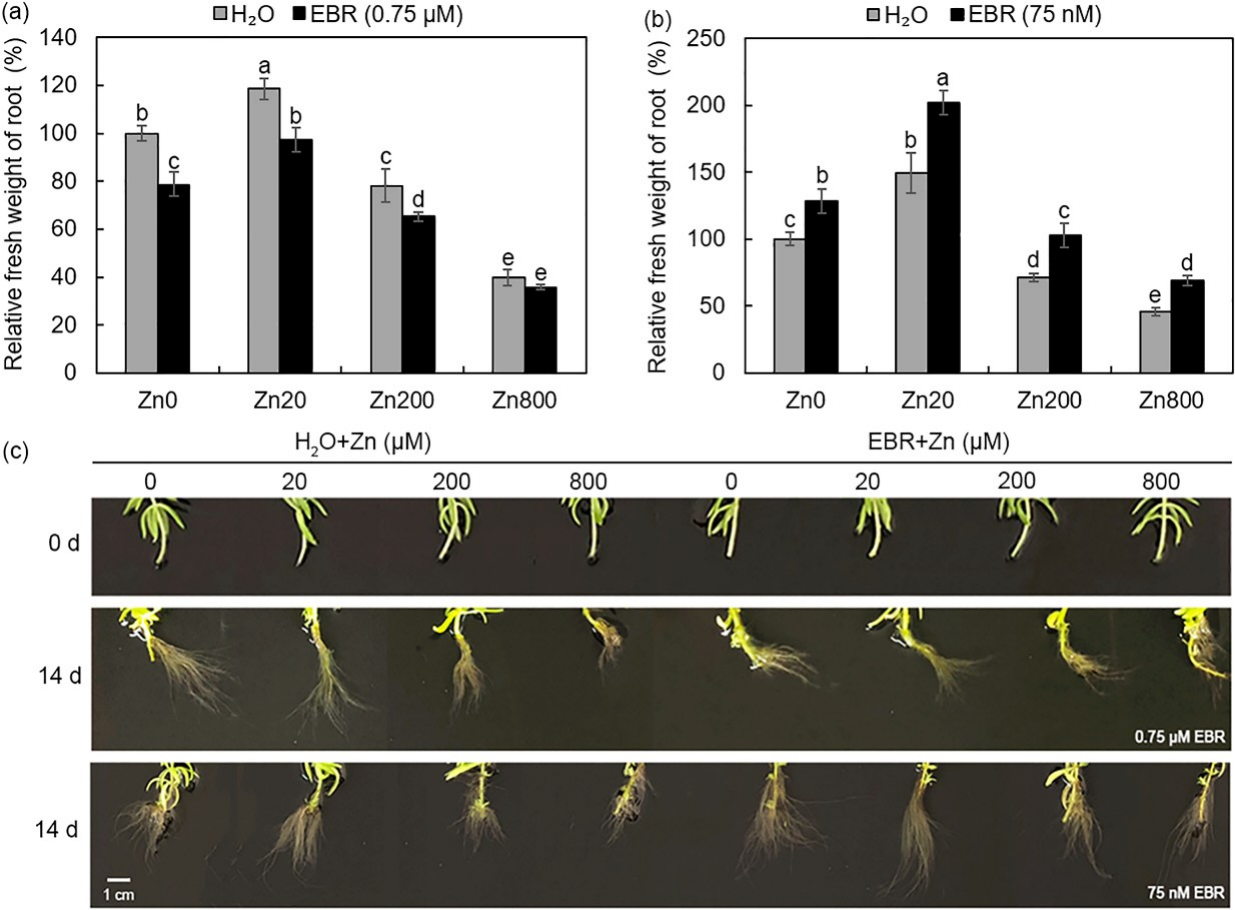
Effects of different concentrations of zinc and EBR on root growth of *S*. *lineare*. **(a)** Relative root fresh weight of *S*. *lineare* treated with 0, 20, 200 and 800 μM ZnSO_4_ and sprayed 0.75 μM EBR for 14 d. **(b)** Relative root fresh weight of *S*. *lineare* treated with 0, 20, 200 and 800 μM ZnSO_4_ and added 75 nM EBR to culture medium for 14 d. **(c)** Photos of *S*. *lineare* root treated with Zn and EBR for 14 d.

We then tested the effect of zinc and EBR on *S*. *lineare* roots. As compared with untreated control plants, Zn20 increased the fresh weight of roots by 19%, while Zn200 and Zn800 decreased the root weight by 22% and 60%, respectively (Fig 2A), in agreement with the previous report that Zn in excess inhibited root growth of *Hordeum vulgare* [36]. Next, we tested whether or not similar protective effects existed for EBR on zinc-stressed roots. Unexpectedly, spraying with 0.75 μM EBR decreased the root fresh weight by ~20% under Zn0, Zn20 and Zn200, but did not significantly affect the root growth under Zn800 (Fig 2A and 2C). Previously, BRs at nM concentrations have been reported to promote the root growth, while BRs at μM inhibited the hypocotyl elongation and primary root growth of wild-type seedlings [37]. Thus, it seemed to be necessary to identify the appropriate EBR concentrations for its optimal effects on root growth. For this purpose, 75 nM EBR was added directly to the culture medium and after 14 days, the root fresh weight was found to be increased by 28–51% at four doses of Zn (Fig 2B and 2C). However, in this study we still used the leaf-spraying method in the following analyses since that it resembled the soil-growing conditions more than hydroponic cultures, and the inhibitory effects of EBR on root growth were fairly moderate at high concentrations of Zn (Fig 2A and 2C).

### EBR decreased the accumulation of H_2_O_2_, O_2_^•−^ and MDA in Zn-stressed *S*. *lineare*

**Fig 3:**
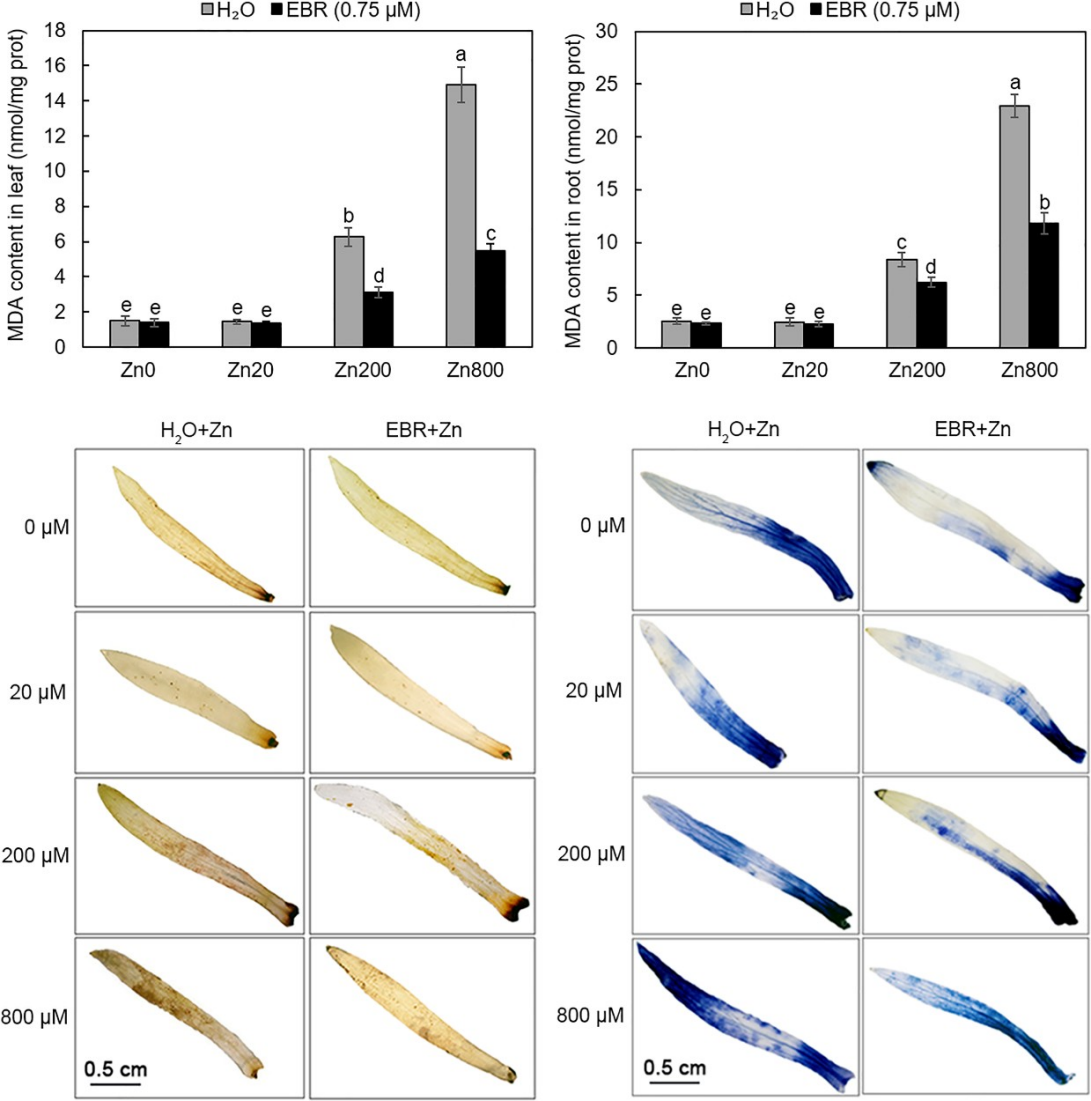
Effect of zinc and EBR on MDA, H_2_O_2_ and O2•^−^ contents of *S*. *lineare*. **(a)** MDA levels in leaves; **(b)** MDA levels in roots; **(c)** H_2_O_2_ accumulation stained with DAB; **(d)** O_2_^•−^ accumulation stained with NBT.

MDA is an indicator of lipid peroxidation of stressed plants [38]. In this study, as zinc concentrations increased, so did the MDA content in leaves and roots. At Zn800, the contents of MDA in leaves and roots were about 10-fold of those in untreated control plants. Application of EBR reduced the MDA accumulation at Zn200 and Zn800 by 103% and 172%, respectively; and in roots by 34% and 94%, respectively (Fig 3A and 3B). Thus, the protective effects of EBR against high dose zinc may be related to the decrease of lipid peroxidation and such mitigation was more prominent in shoots than in roots.

In order to further investigate the association of oxidative damage and Zn toxicity in *S*. *lineare*, we performed histochemical analysis to detect the H_2_O_2_ and O_2_^•−^ accumulation in Zn-treated stems. As expected, Zn-treated tissues exhibited more pronounced spots when compared to control plants but EBR supply obviously decreased the staining intensity (Fig 3C and 3D), indicating that the addition of EBR reduced the generation of Zn stress-induced ROS as previously reported for *Raphanus sativus* [39].

### EBR enhanced the antioxidant enzymatic activities in Zn-stressed *S*. *lineare*

**Fig 4:**
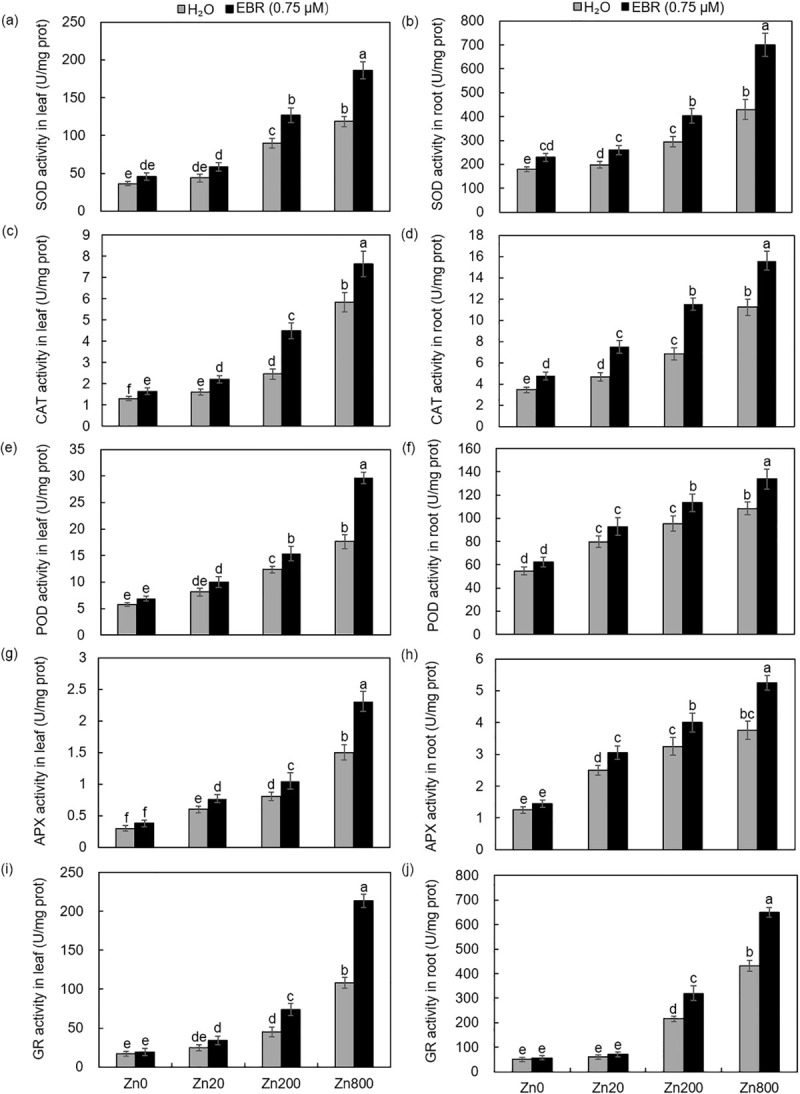
Antioxidant enzymatic activities in leaves and roots of *S*. *lineare* upon Zn and EBR treatments. **(a, b)** SOD activities; **(c, d)** CAT activities; **(e, f)** POD activities; **(g, h)** APX activities; **(i, j)** GR activities.

To survive and adapt to the oxidative damages caused by ROS formation, plants have evolved a defense network that consists of ROS-removing enzymes such as SOD, CAT, POD, APX and GR to maintain the cellular redox homeostasis within certain thresholds [40, 41]. As a main O_2_^•−^ scavenger, the enzymatic action of SOD produces H_2_O_2_ and O_2_ and constitutes the first enzyme defense against ROS. Next, H_2_O_2_ can be broken down into H_2_O and O_2_ by CAT and several peroxidases [42]. In this work, Zn200 stress significantly elevated SOD activities in leaves and roots, and when compared to plants treated by Zn only, concomitant supply of EBR further increased SOD activities by 20–40% under three doses of Zn (0, 20 and 200) and by nearly 60% under Zn800 (Fig 4A and 4B).

Zn treatment also dramatically induced the CAT activity in *S*. *lineare* leaves and EBR application caused further increases. However, unlike SOD, whose activity reached its maximum at Zn800, the induction of CAT by EBR at Zn800 was less pronounced than that at Zn200 (Fig 4C and 4D). As another important indicator of enzymatic antioxidative capacities, the POD activities were increased by 40–207% and by 40–98% in leaves and roots, respectively; when compared to control plants under three doses of Zn, but the effects of EBR on POD activities in both tissues were only minor at three doses of Zn (0, 20 and 200), except that at Zn800, its activity in leaves was significantly further increased by 68% (Fig 4E and 4F).

As the first step of the ascorbate-glutathione cycle, ascorbate peroxidase (APX) plays an important role in reducing the accumulation of H_2_O_2_ by using two molecules of ascorbate to reduce H_2_O_2_ to water [43]. In this study, the activities of APX in leaves and roots were 4- and 2-fold, respectively; of untreated control plants (Fig 4G and 4H). Exogenous addition of EBR to *Solanum lycopersicum* seedlings has been demonstrated to significantly increase the activities of APX under zinc stress [44]. In this work, a 50% increase in APX activity by EBR was observed under Zn800 (Fig 4G and 4H). GR is an important antioxidant enzyme in plants to reduce the oxidized glutathione disulfide (GSSG) into the reduced glutathione (GSH), thus providing another layer of reducing power for the removal of ROS and protects plants from oxidative damages [45]. Among the above-mentioned antioxidant enzymes, GR turned to be the most inducible enzyme by Zn800 in *S*. *lineare* and EBR application further increased its activities by 96% and 50%, respectively; in shoots and in roots at Zn800 (Fig 4I and 4J).

In summary, exogenous application of EBR enhanced the activities of various antioxidant enzymes to alleviate the toxic effects of zinc on *S*. *lineare* with generally more obvious inductions detected in the above-ground part than in the below-ground part (Fig 4).

### EBR enhanced the AsA and GSH contents in Zn-stressed *S*. *lineare*

**Fig 5:**
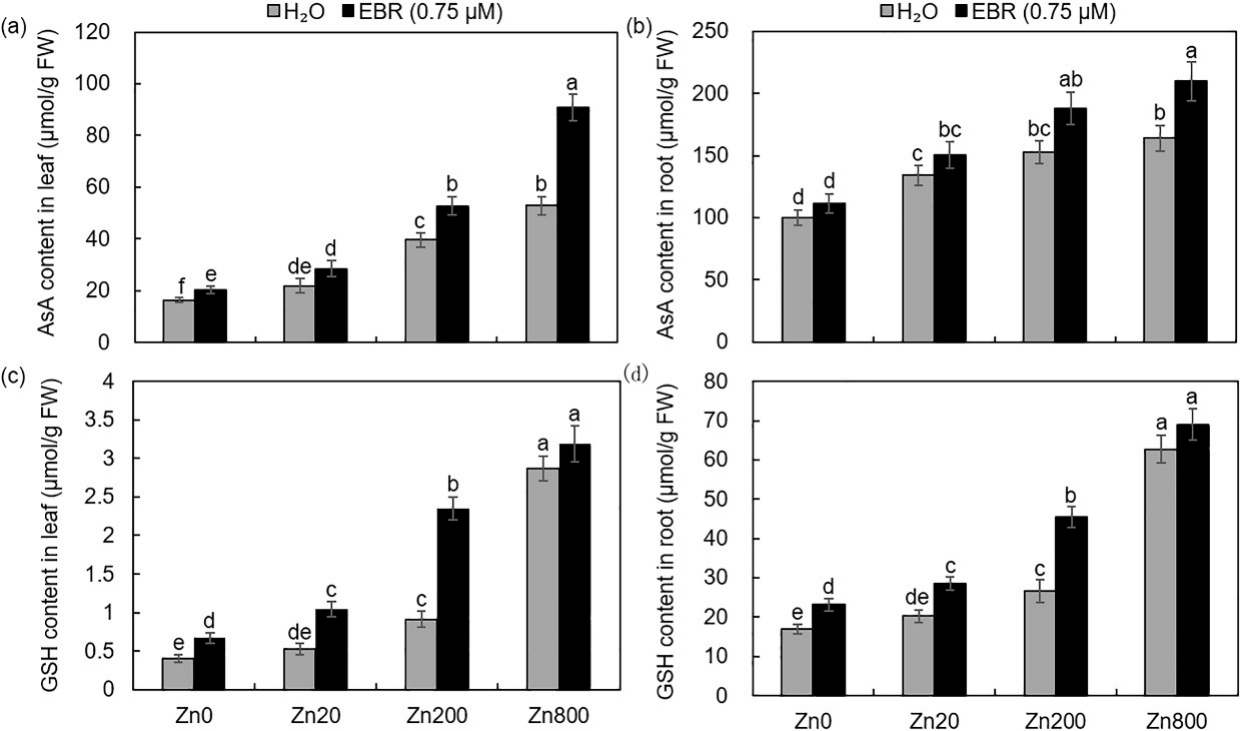
Total AsA and GSH levels in leaves and roots of *S*. *lineare* upon Zn and EBR treatments. **(a, b)** AsA levels; **(c, d)** GSH levels.

AsA and GSH are non-enzymatic antioxidant substances that maintain the balance between ROS production and elimination [46]. With increasing three concentrations of zinc, the AsA contents were increased by 34–222% and 34–64% in *S*. *lineare* leaves and roots, respectively; and EBR supplementation further stimulated the AsA contents in leaves by 24–72%, but had little effects in roots (Fig 5A and 5B). Under three Zn doses (0, 20 and 200), the GSH contents in *S*. *lineare* were elevated by 29–203% shoots and by 19–208% in roots, and EBR application resulted further increased GSH in leaves by 64–156% and in roots by 36–71%, compared to Zn-exposed plants without EBR (Fig 5C and 5D). However, the application of EBR under Zn800 did not significantly change the GSH contents either in leaves or in roots, which was distinct from the case of AsA. Thus, in addition to increasing the activities of antioxidative enzymes, EBR also increased the content of antioxidants agents such as AsA and GSH in *S*. *lineare* to protect plants from the oxidative damage exerted by excessive Zn. Such induction of antioxidants seemed to be dose-dependent and at extremely high concentrations, the accumulation of antioxidants might reach the maximum level and could not be further elevated.

## Conclusion

When compared with other plants reported in literature, *S*. *lineare* showed a relatively higher tolerance to zinc stress, but concentration higher than Zn200 still caused significant growth retardation. When exogenously applied, the phytohormone EBR showed obvious growth-enhancing effects on *S*. *lineare* at all tested Zn doses (0, 20, 200 and 800). Under Zn800, EBR had the most significant effects as the stem length, fresh weight and internode length were increased by 111%, 85% and 157%, respectively. Such protective effects of EBR have been achieved through decreasing the internal accumulation of ROS, which was manifested by the elevated antioxidant activities and the upregulated accumulations of antioxidant agents. Although the exact molecular mechanism underlying the protection of EBR against Zn toxicity remains largely unknown due to the absence of genomic sequence data of *S*. *lineare*, the growth promotion of BR phytohormones on *Crassulaceae* plants may imply a simple method to increase the planting range of similar pioneer plants in heavy metal contaminated areas.
